# N‑Heterocyclic Carbene-Ag(I)-Phosphine Complexes: Comprehensive Synthesis, Characterization, and Bonding Analysis via Density Functional Theory

**DOI:** 10.1021/acsomega.5c07921

**Published:** 2026-02-20

**Authors:** Abdollah Neshat, Mohammad Reza Yousefshahi, Mahdi Cheraghi, Vaclav Eigner, Michal Dusek

**Affiliations:** † Department of Chemistry, 113403 Institute for Advanced Studies in Basic Sciences (IASBS), 444 Prof. Sobouti Blvd, Gava Zang, Zanjan 45137-66731, Iran; ‡ 86889 Institute of Physics of the Czech Academy of Sciences, Na Slovance 2, Prague 8 18221, The Czech Republic

## Abstract

The substitution of a chloro ligand in (IPr)­Ag–Cl with aliphatic and aromatic phosphine ligands, PPh_3_, PCy_3_, PPh_2_Py, dppf, dppm, and dppe, was investigated. Unlike gold­(I) complexes, the heteroleptic complexes, [(IPr)­Ag-PR_3_]^+^, are less stable in the solution phase. The order of mixing reactants, solvent type, and reaction time plays crucial roles in obtaining the desired product. In a methanolic solution, the generated [(IPr)­Ag­(I)]^+^ ions from (IPr)­Ag–Cl are less stable compared with [(IPr)­Au­(I)]^+^ and only PCy_3_ and PPh_2_Py stabilize this cationic fragment in solution. In the single-crystal X-ray diffraction analysis of complexes **1** and **2**, the Ag­(I) centers exhibited a linear geometry. Other forms of silver­(I) complexes containing only phosphine ligands were also formed in a solution containing the (IPr)­Ag–Cl precursor. Computational analysis of the complexes using density functional methods provided insights into the nature of electronic transitions, noncovalent interactions, and fragmental bonding contributions.

## Graphical abstract

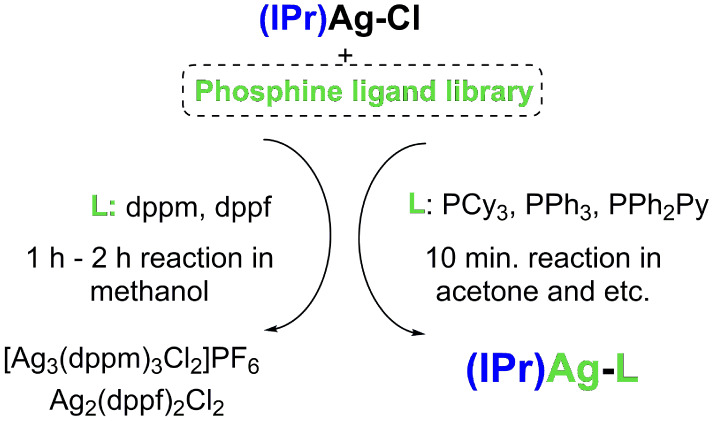

## Introduction

N-heterocyclic carbene (NHC) ligands and their transition metal complexes have been at the forefront of advancements in molecular catalysis and bioinorganic chemistry research in recent years.^1–31^ Similar to phosphine ligands, this class of ligands can provide excellent thermodynamic stability when they coordinate with transition metal ions. Despite the extensive literature on the synthesis and coordination chemistry of NHC ligands, significant potential remains for further investigation into the synthesis of certain classes of transition metal complexes—such as those involving group 11 metal ions—that incorporate these ligands. Notable among them are the silver-NHC complexes, which also include a different second ligand at the silver center, forming heteroleptic complexes. For example, silver­(I) complexes with N-heterocyclic carbenes (NHC) and a labile halogen ligand have been employed as transmetalation agents for the synthesis of a wide range of transition metal complexes, such as those involving gold­(I) and copper­(I).^11,32–38^ However, a literature search reveals that our understanding of the synthesis of heteroleptic silver­(I) complexes, [(NHC)­Ag-X; X = heteroatom from donor ligand], which contain an NHC and a different type of ligand, as well as their stability and applications, is limited.^39^ The known examples of this class of silver complexes have already demonstrated significant utility in catalysis and medicinal chemistry as anticancer and antibacterial agents.^28,38,40–48^ While heteroleptic silver­(I) complexes of NHC ligands with bidentate nitrogen and phosphorus ligands have been reported,^28,40,44,47^ to the best of our knowledge, there is only one description of such complexes with monodentate phosphine ligands, in which the reactivity of [(IPr)­Ag­(NH_3_)]^+^ has been utilized as a stable species to prepare heteroleptic [(IPr)­Ag­(phosphine)]^+^ complexes.^49^ We have recently shown that (IPr)­Au–Cl is a versatile precursor for the synthesis of heteroleptic gold­(I) complexes such as [(IPr)­Au­(I)-PR_3_]^+^ and (IPr)­Au–S­(S)­P­(OR)_2_ with phosphine and sulfur donor ligands.^5,6^ Similarly, (IPr)­Ag–Cl can also be utilized to generate (IPr)­Ag–S­(S)­P­(OR)_2_ complexes by simply reacting it with dialkyl dithiophosphates.^5^ To determine whether the procedure implemented for gold­(I) complexes can be extended to silver complexes with N-heterocyclic carbene (NHC) and phosphine ligands, we investigated the synthesis of heteroleptic silver­(I) complexes containing an NHC ligand, such as 1,3-Bis­(2,6-isopropylphenyl)-imidazol-2-ylidene, and common phosphine donors like PR_3_ (where R represents alkyl or aryl groups). Other than providing a straightforward procedure for the synthesis of [(IPr)­Ag­(I)-PR_3_]^+^ type complexes, we aimed to compare the stability of these heteroleptic silver­(I) and gold­(I) complexes and to address key electronic and steric parameters. The knowledge gained regarding the stability or instability, as well as the types of species that could be formed upon dissolution of a metal complex in solution, could provide significant insights into the nature of these species when they are utilized as antitumor agents or catalysts in organic transformations.

## Results and Discussion

As depicted in Scheme 1, 1,3-bis­(2,6-isopropylphenyl)-imidazol-2-ylidene-silver­(I) chloride, (IPr)­Ag–Cl, serves as the synthon for the derivatization reactions. It turns out that, unlike the synthesis of [(IPr)­Au­(I)-PR_3_]^+^ (R: alkyl, aryl) complexes,^6^ the reaction time plays a crucial role in the stability of the intermediates and the overall yield of the reaction for the synthesis of silver­(I) counterparts, i.e., [(IPr)­Ag-PR_3_]^+^ (R: alkyl, aryl). We also observed that the sequence of reactant addition is crucial to the outcome of the reaction. Additionally, the synthesized [(IPr)­Ag-PR_3_]^+^ complexes are more prone to decomposition in solution.

**Scheme 1:**
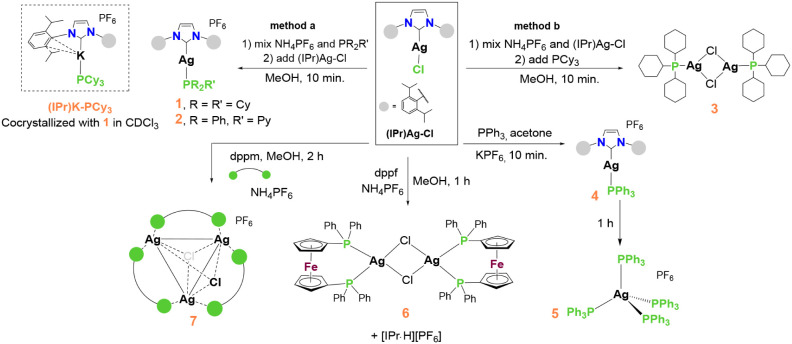
Reaction Conditions for the Synthesis of 1-7; Method a: Phosphine Ligand (PR_2_R’) and NH_4_PF_6_ are Mixed First, Followed by the Addition of (IPr)­Ag–Cl; Method b: NH_4_PF_6_ and (IPr)­Ag–Cl are Reacted First, Followed by the Addition of PCy_3_

The derivatization reaction of (IPr)­Ag–Cl with PCy_3_ and PPh_2_Py in methanol, initiated by dissolving the NH_4_PF_6_ and phosphine ligand in methanol (method a), produced the desired products **1** and **2** within 10 min. If the sequence of addition of the reactants is changed, that is, if (IPr)­Ag–Cl and NH_4_PF_6_ are reacted first, and then the reaction is continued by adding the PCy_3_ ligand (method b), the major metal complex characterized is **3**, which is a bimetallic silver­(I) complex featuring bridging chloro and terminal PCy_3_ ligands (CCDC deposition number 1271262). The literature procedure for synthesizing complex **3** involves the reaction of silver chloride with PCy_3_ in pyridine.^50^

The reaction of (IPr)­Ag–Cl with NH_4_PF_6_ in methanol, followed by the addition of PPh_3_, primarily yielded [IPr·H]­[PF_6_], with no formation of the desired product, [(IPr)­Ag-PPh_3_]^+^. After workup of the reaction, clear crystals were formed, and their crystallography revealed them to be [IPr·H]­[PF_6_] (crystal data matched a previously published structure with the CCDC deposition number 1408942). We speculate that this occurs via initial decoordination of the chloride ligand and formation of [(IPr)­Ag]­[PF_6_] (eq 1). This is a salt metathesis reaction in which a coordinating counterion is replaced by a noncoordinating counterion. However, the product of this salt metathesis reaction is not stable in a protic solvent such as methanol, resulting in the formation of the protonated IPr ligand, [IPr·H]^+^, which is stabilized by the PF_6_^–^ anion. It is notable that the presence of coordinated chloro anions in **3** indicates that NH_4_^+^ cation could as well be the protonating agent. In eq 1, the carbene ligand, which is weakly coordinated to the silver metal in [(IPr)­Ag]­[PF_6_], can be protonated in the presence of methanol. This reaction occurs rapidly, and the addition of triphenylphosphine is insufficient to stabilize these monocoordinated species. Unlike [(IPr)­Ag]­[PF_6_], [(IPr)­Au]­[PF_6_] is stable in methanol, and its reaction with phosphine has been reported.^6^ This observation is consistent with the calculated higher bond energies of NHC with gold­(I) ions compared to silver­(I) ions.^51^ It is notable that the reaction of a free carbene with methanol or the methoxide anion is well understood.^52^

1 $$\left(\mathrm{IPr}\right)\mathrm{Ag}‐\mathrm{Cl}\overset{{\mathrm{NH}}_{4}{\mathrm{PF}}_{6}}{\underset{{\mathrm{MeOH},\mathrm{PPh}}_{3}}{\to }}\left[\left(\mathrm{IPr}\right)\mathrm{Ag}\right]\left[{\mathrm{PF}}_{6}\right]{+\mathrm{NH}}_{4}\mathrm{Cl}+\mathrm{free}\;{\mathrm{PPh}}_{3}$$

Reducing the reaction time to 10 min instead of 1 h in methanol did not alter the outcome. Notably, after 5 min, a color change from clear to light gray is observed upon the addition of NH_4_PF_6_ or KPF_6_ to (IPr)­Ag–Cl in methanol, which we speculate is due to the formation of silver oxide. This observation demonstrates that [(IPr)­Ag­(I)]^+^, unlike [(IPr)­Au­(I)]^+^, is more susceptible to decomposition in a polar medium. Therefore, the addition of the phosphorus ligand should occur immediately after the addition of the NH_4_PF_6_. Changing the sequence of reactant addition by first dissolving the phosphine ligand and NH_4_PF_6_, followed by the addition of the silver­(I) precursor, did not alter the outcome in this case. A similar reaction conducted in acetone in 10 min, where PPh_3_ and KPF_6_ were dissolved first, followed by the addition of (IPr)­Ag–Cl, yielded the desired product, **4**, as indicated by the ^1^H NMR spectrum. However, the crystallization of the reaction solution produced two distinct types of crystals: clear crystals identified as [IPr·H]­[PF_6_] (CCDC deposition number 1408942) and yellow crystals that did not yield a solvable diffraction pattern. Interestingly, extending the reaction time to 1 hour resulted in clear light yellow crystals in a mixture of CDCl_3_ and diethyl ether, which were characterized as tetrahedral Ag­(PPh_3_)_4_·PF_6_, **5**, with the crystal structure depicted in Figure 3. Comparing synthesis of complexes **1**-**4** in methanol, demonstrates that PCy_3_ and PPh_2_Py stabilize the [(IPr)­Ag­(I)]^+^ fragment more effectively than PPh_3_. Furthermore, the type of phosphine, whether aliphatic or aromatic, significantly impacts its coordination to the silver­(I) center and the stability of the resulting heteroleptic NHC-Ag-PR_3_ complex. Derivatization of (IPr)­Ag­(I)-Cl with bidentate phosphine donors such as dppf, dppm, and dppe was conducted in methanol. The single crystal structure of the product resulting from the reaction with the dppe ligand in methanol or acetone matched the known compound [IPr_2_·Ag]­PF_6_ (CCDC deposition number 1518573). The reaction with dppf, after 10 min, primarily yielded [IPr·H]­[PF_6_], as indicated by the single crystal structure obtained from a mixture of chloroform and *n*-hexane. Extending the reaction time to 1 h and following a similar workup, along with subsequent crystallization in chloroform and ether, produced both large and small crystals that were characterized as known structures [IPr·H]­[PF_6_] and complex **6** (in Scheme 1), uploaded to CCDC with deposition numbers 1408942 and 1314548.^53^ The ^1^H NMR spectrum of the reaction mixture also confirms the presence of these species, along with [IPr_2_·Ag]­PF_6_ (see signals marked with stars in Figure S10). Unlike their gold­(I) counterparts, and based on observations from reactions leading to complexes **5** and **6**, it can be concluded that both PPh_3_ and dppf are unsuitable phosphine donors for stabilizing the [IPr-Ag]^+^ fragment. The reaction with dppm in methanol, as shown in Scheme 1, predominantly produced a trimetallic silver­(I) complex featuring bridging chloro and dppm ligands (**7**). Literature procedures for cationic trimetallic silver­(I) complexes with differing anions typically involve the reaction of silver nitrate with dppm ligands in acetonitrile and methanol.^54^ A summary of the key findings in the synthesis of the complexes depicted in Scheme 1, which illustrates how reaction time, the sequence of reactant addition, and solvent choice influence the final product, is provided in Figures 1 and 2.

**Figure 1:**
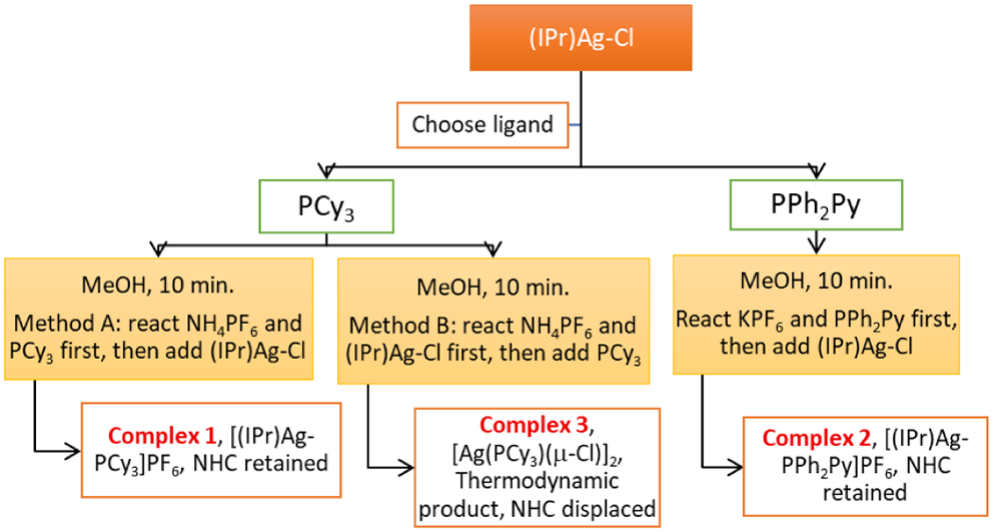
Flowchart of the syntheses of **1–3** summarizing reaction time, sequence of reactant addition, and solvent choice.

**Figure 2:**
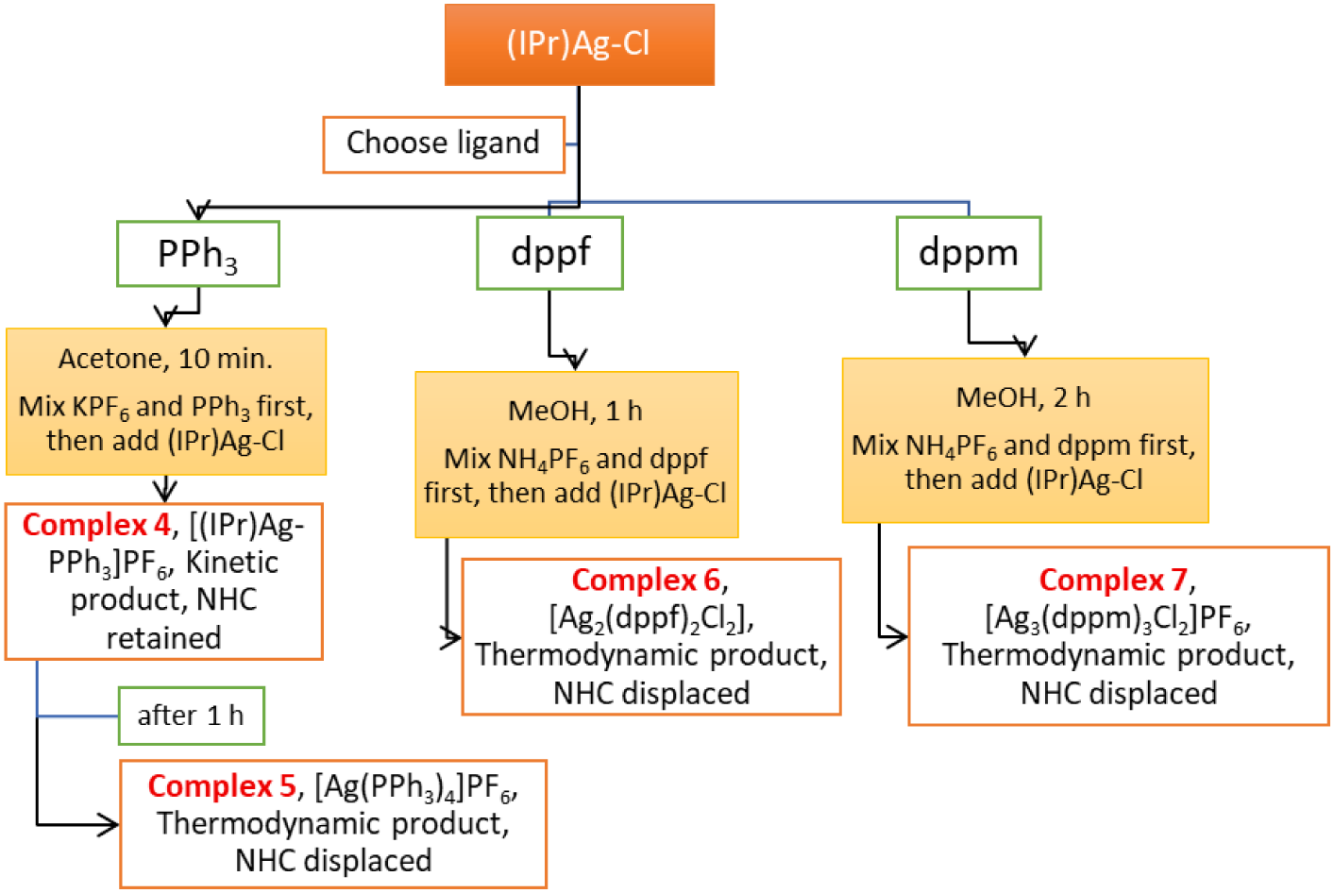
Flowchart of the syntheses of **4–7** summarizing reaction time, sequence of reactant addition, and solvent choice.

The^1^H NMR spectrum of compound **1** (Figure S1) displays signals for eight aromatic protons in the range of 7.58 to 7.14 ppm. The aliphatic proton signals appear as multiplets in the range of 1.71 to 0.79 ppm, with integration matching that of 57 protons, further confirming the synthesis of this complex. The ^13^C NMR spectrum of compound **1** shows evidence of carbon–silver coupling. The measured coupling constants of ^1^ *J*(^13^C–^107^Ag) and ^1^ *J*(^13^C–^109^Ag) are 240 and 270 Hz, respectively, which fall within the range of values reported for NHC-Ag­(I) complexes.^55^ The ^31^P NMR spectrum of compound **1** (Figure S3) also indicates phosphorus–silver coupling, with measured coupling constants of ^1^ *J*(^31^P–^107^Ag) and ^1^ *J*(^31^P–^109^Ag) at 534 and 470 Hz, respectively, consistent with values reported for phosphine–silver complexes.^50,54^

In contrast to complex **1**, the ^13^C NMR spectrum of **2** (Figure S5) does not exhibit the anticipated downfield C–Ag coupling. Additionally, the ^31^P NMR spectrum of **2** (Figure S6) reveals a high-field shifted broad signal centered at approximately 17.2 ppm. This is the result of fast exchange of coordinated phosphine ligand. The reaction leading to complex **3** was characterized using ^1^H NMR spectroscopy (Figure S7). In the ^1^H NMR spectrum of compound **4** (Figure S8), aryl protons appear as multiplets in the range of 7.64–6.97 ppm, while the diagnostic isopropyl signals are observed as two overlapping doublets in the range of 1.30–1.20 ppm, with peak integration corresponding to the expected 24 protons. When the reaction time for the synthesis of compound **4** is set to 1 h, an increase in the signal intensity of free [IPr·H]­[PF_6_] is noted, clearly indicating its decomposition. The ^31^P NMR spectrum of complex **4** (Figure S9) displays multiple unresolved signals between 19 and 10.7 ppm, which, unlike the phosphorus NMR signal of complex **1**, is not well resolved. A triplet signal with a coupling constant of 972 Hz was also observed at −18.2 ppm, the origin of which remains unidentified. Since the ^1^H NMR spectrum of this compound shows no signs of degradation or free ligand, the broadening can also be attributed to fast phosphine exchange in the solution phase of **4**. While the ^1^H NMR spectrum of complex **4** supports its proposed structure in Scheme 1, the presence of additional signals in the phosphorus spectrum (which requires further scan time) clearly indicates the instability of this complex in the solution phase. Obtaining a clear ^13^C NMR spectrum of these complexes requires even more scan time; therefore, due to the observed instability in solution, the ^13^C NMR spectrum of complex **4** was not recorded.

### X-ray Crystallography of **1**, **2**, **5**, and **7**

In four cases, single-crystal X-ray crystallography studies on suitable crystalline samples provided complete structural data. The slow diffusion of *n*-hexane into a deuterated chloroform solution of compound **1** at 0 °C yielded prism-like colorless crystals, which were analyzed using X-ray diffraction. Single crystals of compound **2** were obtained in a similar manner, and the resulting colorless crystals also exhibited a prism shape. The molecular structures of compounds **1**, **2**, and **5** are depicted in Figure 3, and the selected structural parameters are summarized in Table S1. The molecular structure of complex **7** is depicted in Figure 4. Complex **1** crystallized in the orthorhombic *Pca*2_1_ space group, with the silver (I) center adopting a nearly linear geometry, characterized by a C2–Ag1–P30 angle of 179.11(17)°. As shown in Figure 3, the asymmetric unit of compound **1** also includes a cocrystallized potassium complex, (IPr)­K-PCy_3_, which we speculate is formed during the crystallization process as a result of the transmetalation of silver with potassium carbonate, used as a stabilizing agent for deuterated solvents such as CDCl_3_.^56^ This observation demonstrates a straightforward method for obtaining alkali metal heteroleptic complexes through a simple transmetalation reaction with silver complexes. Similar to the silver ion, the potassium metal center in compound **1** also adopts a linear geometry with N-heterocyclic carbene (NHC) and tricyclohexylphosphine (PCy_3_) ligands. However, a third coordination from the *ipso*-carbon atom of the nearby 2,6-diisopropylphenyl ring is also observed, which is typical of potassium π-aryl systems.^57^

**Figure 3:**
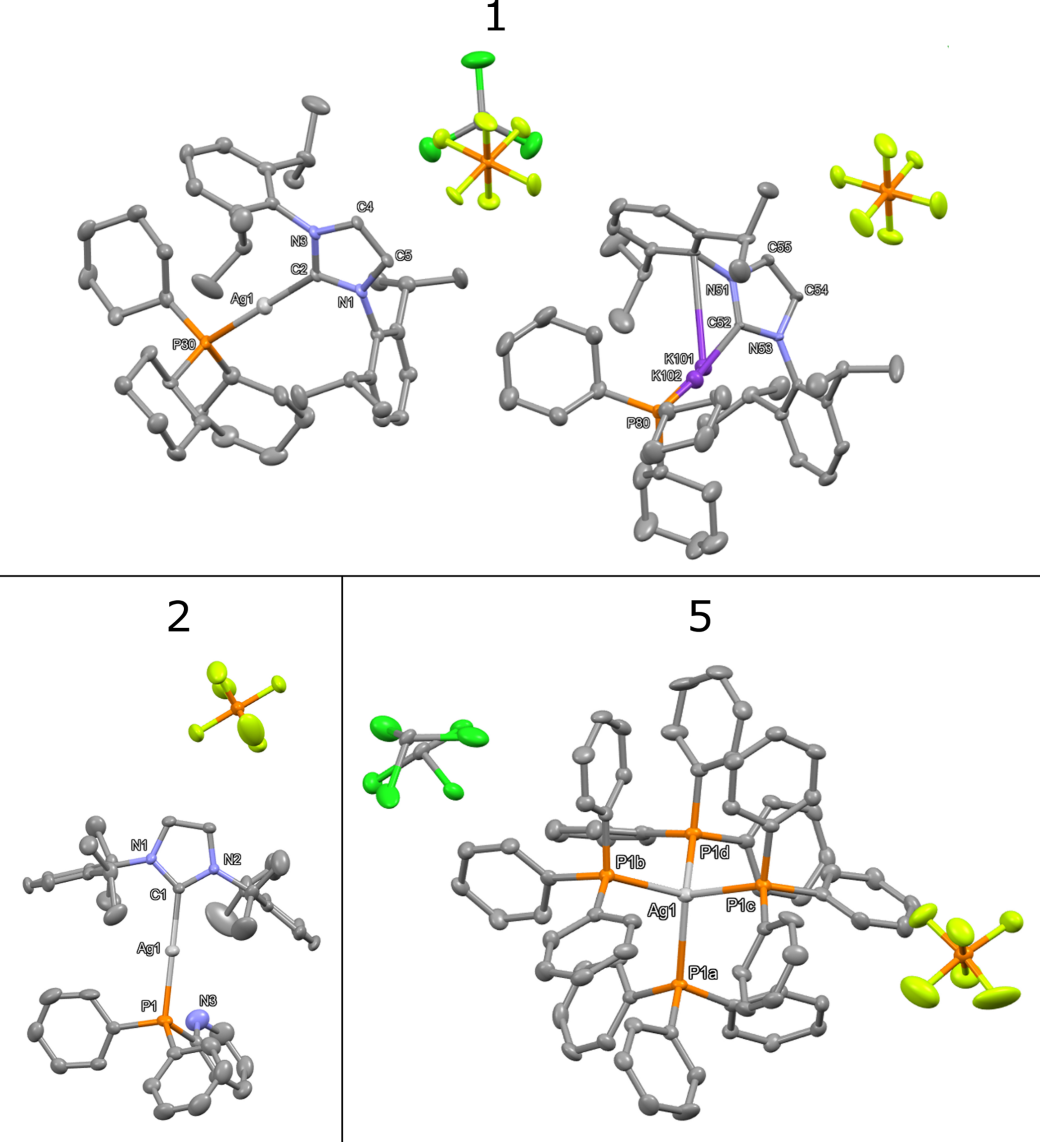
X-ray crystal structure of **1** (top), **2** (bottom left), and **5** showing thermal ellipsoids for non-hydrogen atoms at 30% probability level. Hydrogen atoms are omitted for clarity. Selected bond lengths [Å], bond angles [°] (**1**): C2–Ag1 2.062(6), P30–Ag1 2.3449(17), C52–K101 2.032(6), P80–K102 1.503(5), C2–Ag1–P30 179.11(17), C52–K101–P80 176.43(18). (**2**): Ag1–P1 2.3518(8), Ag1–C1 2.094(2), P1–Ag1–C1 175.87(9). (**5**): Ag1–P1a 2.6146(10), Ag1–P1b 2.6389(10), Ag1–P1c 2.6448(10), Ag1–P 1d 2.6270(10), P1a–Ag1-P1b 108.96(3), P1a–Ag1-P1c 110.73(3), P1a–Ag1-P 1d 109.32(3), P1b–Ag1-P1c 108.04(3), P1b–Ag1-P 1d 110.70(3), and P1c–Ag1-P 1d 109.07(3).

**Figure 4:**
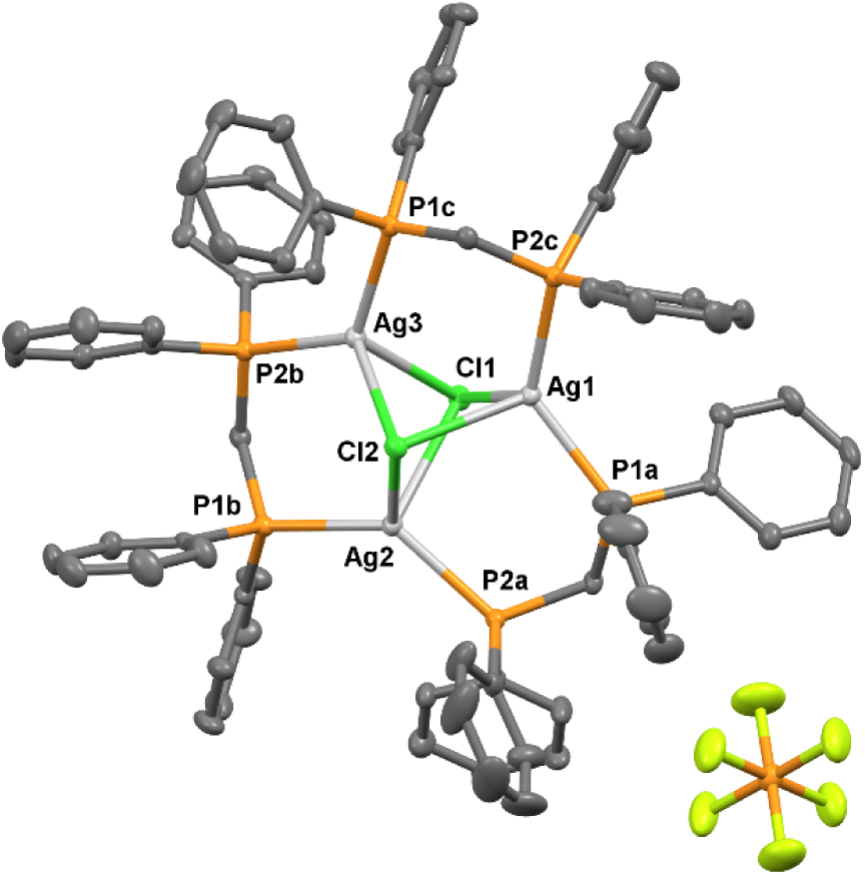
X-ray crystal structure of **7** showing thermal ellipsoids for non-hydrogen atoms at 30% probability level. Hydrogen atoms are omitted for clarity. Selected bond lengths [Å], bond angles [°]: Ag1–Cl1 2.6572(9), Ag1–Cl2 2.7610(8), Ag1–P1a 2.4438(9), Ag1–P2c 2.4339(11), Ag2–Cl1 2.7559(9), Ag2–Cl2 2.7116(9), Ag2–P2a 2.4534(9), Ag2–P1b 2.4575(10), Ag3–Cl1 2.7414(7), Ag3–Cl2 2.6650(9), Ag3–P2b 2.4398(10), Ag3–P1c 2.4418(11), Cl1–Ag1–Cl2 86.56(3), P1a–Ag1–P2c 125.19(3), Cl1–Ag2–Cl2 85.61(3), P2a–Ag2–P1b 133.52(4), Cl1–Ag3–Cl2 86.81(2), P2b–Ag3–P1c 126.53­(3), Ag1–Cl1–Ag2 82.28(2), Ag1–Cl1–Ag3 75.23(2), Ag2–Cl1–Ag3 77.050(19), Ag1–Cl2–Ag2 81.21(2), Ag1–Cl2–Ag3 74.78(2), and Ag2–Cl2–Ag3 79.11(2).

Complex **2** was crystallized in the tetragonal *P*4_3_ space group. As shown in Figure 3, the C1–Ag1–P1 angle is less linear (175.87(9)°) compared to that of complex **1**, which can be attributed to the presence of weak hydrogen bonding interactions with nearby hydrogens from isopropyl substituents. A more pronounced form of such interactions has been observed in the [NHC-Au-PPh_2_Py]^+^ complex.^6^

Despite the formation of complex **4** in acetone, the generated complex exhibits limited stability in acetone, leading to the decomposition of complex **4** into complex **5** over a period of 1 h. This is not surprising, given the stability and rich coordination chemistry of silver­(I) ions in the presence of phosphine ligands. The relationship between the type of silver­(I) complex with phosphine donors and the nature of the phosphine ligands, reactant stoichiometry, or even the nature of the anions has been previously investigated in great detail.^58,59^ The minimum and maximum Ag–P bond distances in complex **5** range from 2.6146(10) for the Ag1–P1a bond to 2.6448(10) for the Ag1–P1c bond, which falls within the range reported for [Ag­(PPh_3_)_4_]­SbF_6_.^60^ The bond angles of P1a–Ag1–P1b (108.96(3)), P1a–Ag1–P1c (110.73(3)), P1a–Ag1–P1d (109.32(3)), P1b–Ag1–P1c (108.04(3)), P1b–Ag1–P1d (110.70(3)), and P1c–Ag1–P1d (109.07(3)) are close to the ideal tetrahedral angles and are similar to those found in [Ag­(PPh_3_)_4_]­SbF_6_. Similar attempts to synthesize the [NHC-Ag­(I)-dppm]^+^ complex yielded complex **7**, which is a trinuclear silver­(I) complex with two chloro and three dppm bridging ligands. A change in the sequence of the addition of reactants did not yield the desired heteroleptic [(IPr)­Ag­(I)-dppm]^+^ complex.

### Theoretical Studies

Computational investigations were conducted on complexes **1**, **2**, and **4**. The first step involved optimizing the geometries of the complexes in the gas phase. Table 1 presents a comparison of the specified bond lengths and bond angles of the computed complexes with those obtained from experimental data. The extended transition state—natural orbitals for chemical valence (ETS-NOCV)^61,62^ and reduced density gradient (RDG)^63,64^ analyses were carried out at PBE1PBE­(PBE0)–D3­(BJ)/def2-TZVP (Ag)/def2-SVP^65–68^ (main elements) using Gaussian 09 rev.D01 ^69^ and Multiwfn software.^70^

**Table 1:** Experimental and Calculated Bond Length and Angle of Studied Complexes

Complex	Bond length and angle	Expt.	Calc.
L–M–PCy_3_	Ag–P	2.345	2.368
	Ag–C_carbene_	2.061	2.082
	ZC–Ag–P	179.12	175.28
	Au–P	2.285	2.314
	Au–C^carbene^	2.045	2.037
	ZC–Au–P	174.84	176.70
L–M–PPh_2_Py	Ag–P	2.352	2.361
	Ag–C^carbene^	2.094	2.079
	ZC–Ag–P	175.87	171.81
	Au–P	2.286	2.301
	Au–C^carbene^	2.048	2.029
	ZC–Au–P	177.15	176.51

According to the data presented in Table 1, which compares [(NHC)­Au­(I)-PR_3_]^+^ and [(NHC)­Ag­(I)-PR_3_]^+^, NHC gold­(I) ions form stronger interactions with phosphorus and carbene carbon donor atoms than silver­(I) ions. This indicates a stronger interaction between these atoms, thereby elucidating the significant difference in the ETS-NOCV values between the gold and silver complexes.

As anticipated, similar to our earlier analysis of gold­(I) complexes,^6^ compound **1** exhibits the largest HOMO–LUMO energy gap at 6.148 eV, while compound **2** has the smallest gap at 5.611 eV (Figure 5). All silver and gold complexes, with the exception of the L–M–PPh_3_ complexes, show comparable HOMO and LUMO energy values, and their energy differences (Δ*E*) are similarly comparable. The HOMO primarily extends over the IPr ligand, except in compound **4**, where it also extends over the silver and PPh_3_ ligands (Figure 6). In contrast, the lowest unoccupied molecular orbital (LUMO) is delocalized over the PR_3_ ligands; however, in complex **1**, the LUMO spans the IPr ligand, as well as the silver and phosphorus atoms. The orbital delocalization index has been provided in Figure 6. A lower ODI indicates a greater degree of orbital delocalization and vice versa. The LUMO of **4** has the highest orbital delocalization (4.66), while that HOMO of **2** is the lowest (11.26), mainly localized on aryl wing of IPr.

**Figure 5:**
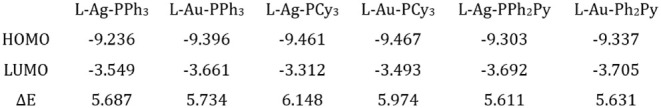
Energy of frontier orbitals of **1**, **2**, **4** and comparison with gold­(I) counterpart. Calculated at the following level of theory: BE1PBE­(D3BJ)/Def2-TZVP (M)/Def2-SVP (rest of the elements).

**Figure 6:**
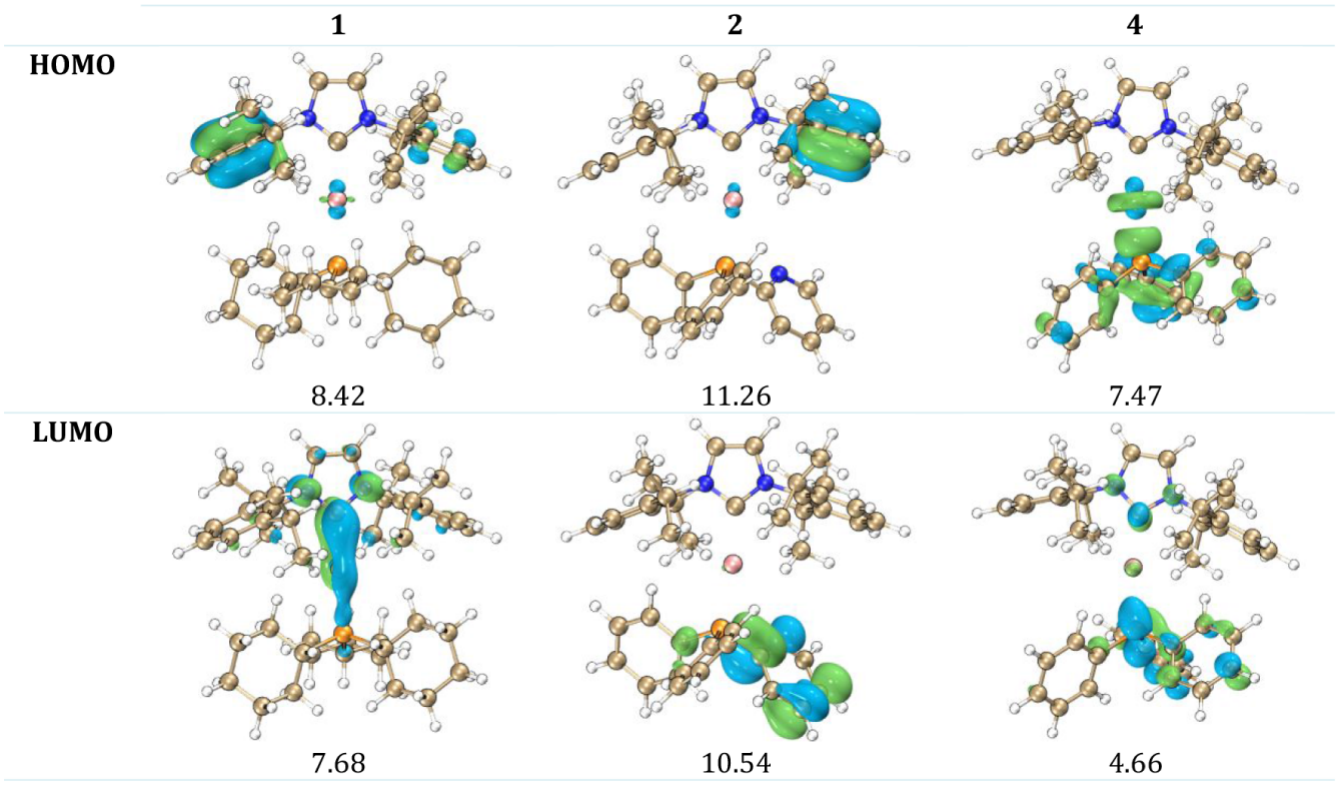
Frontier orbitals of **1**, **2**, and **4** with their ODI. Isosurfaces obtained by following level of theory: PBE1PBE­(D3BJ)/Def2-TZVP (M)/Def2-SVP (rest of the elements) – gas phase.

### RDG Analysis

All three complexes exhibit distinct noncovalent interactions, as illustrated in Figure 7. These interactions include hydrogen bonds, CH···π interactions, CH···CH interactions, Ag···H interactions, and H···π interactions. Additionally, the nitrogen atom of the pyridine substituent interacts with the hydrogen atom attached to isopropyl (IPr). Steric repulsions are also evident, indicated by the orange and red colors. In complex **1**, however, Ag···H interactions are particularly abundant due to the proximity of cyclohexyl hydrogens to the silver atom, in contrast to complexes **2** and **4**.

**Figure 7:**
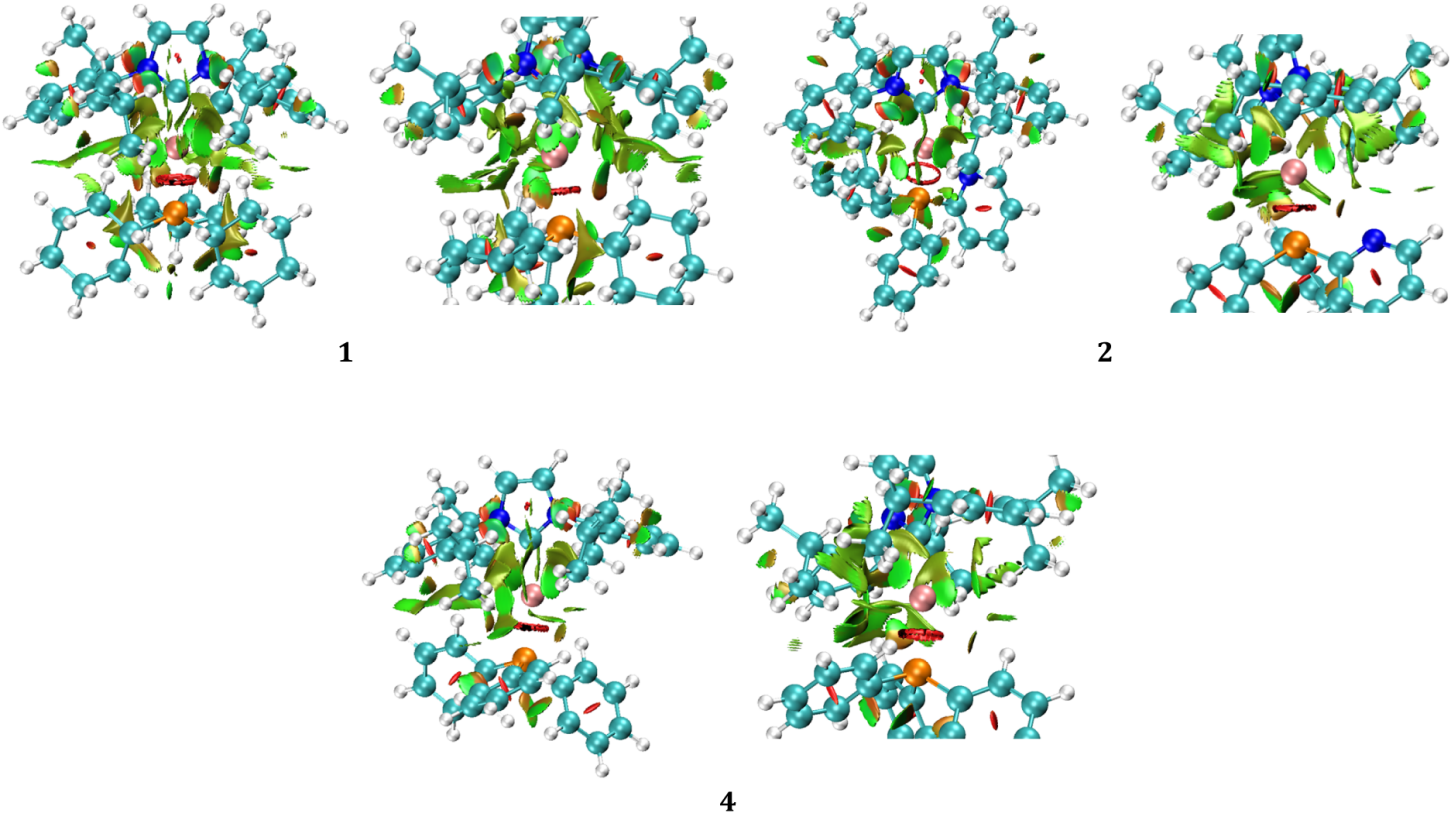
Non covalent interactions (mainly, Ag···H) in **1**, **2**, and **4**. Color code for atoms: orange for P, cyan for C, pink for Ag. The red and orange colors indicate steric repulsions. Green color indicates weak attractions.

### ETS-NOCV Analysis

The Extended Transition State-Natural Orbitals for Chemical Valence (ETS-NOCV) method was employed to gain insights into orbital interactions. According to Table 2, **1** exhibits the highest stabilization energy (−60.32 kcal/mol) resulting from orbital interactions, while **4** shows the lowest stabilization energy (−56.94 kcal/mol). The trend in orbital stabilization energy is as follows: is **1** > **2** > **4**, which aligns with the sequence observed in our previous study for Au: L–Au–PCy_3_ > L–Ag–PPh_2_Py > L–AuPPh_3_ (see Table 2). The primary contributor to Δ*E*_orb_ is the sigma-type interaction (from PR to Ag), as anticipated. The secondary contributor is the π-type interaction (back-donation from Au to the σ* orbital of the P–C bond in PR_3_). Visual inspection reveals significant charge transfer from the blue region, where electron density decreases, to the green region, where electron density increases. The eigenvalues indicated on the arrows represent the number of electrons transferred through orbital interactions, demonstrating that the values for complexes **1** and **2** are similar and approximately four times greater than the eigenvalues associated with back-donation. This suggests that back-donation is not particularly significant in any of the three complexes. It is noteworthy that the Δ*E*_orb_ of Au complexes is nearly twice that of Ag complexes (Table 3).

**Table 2:**
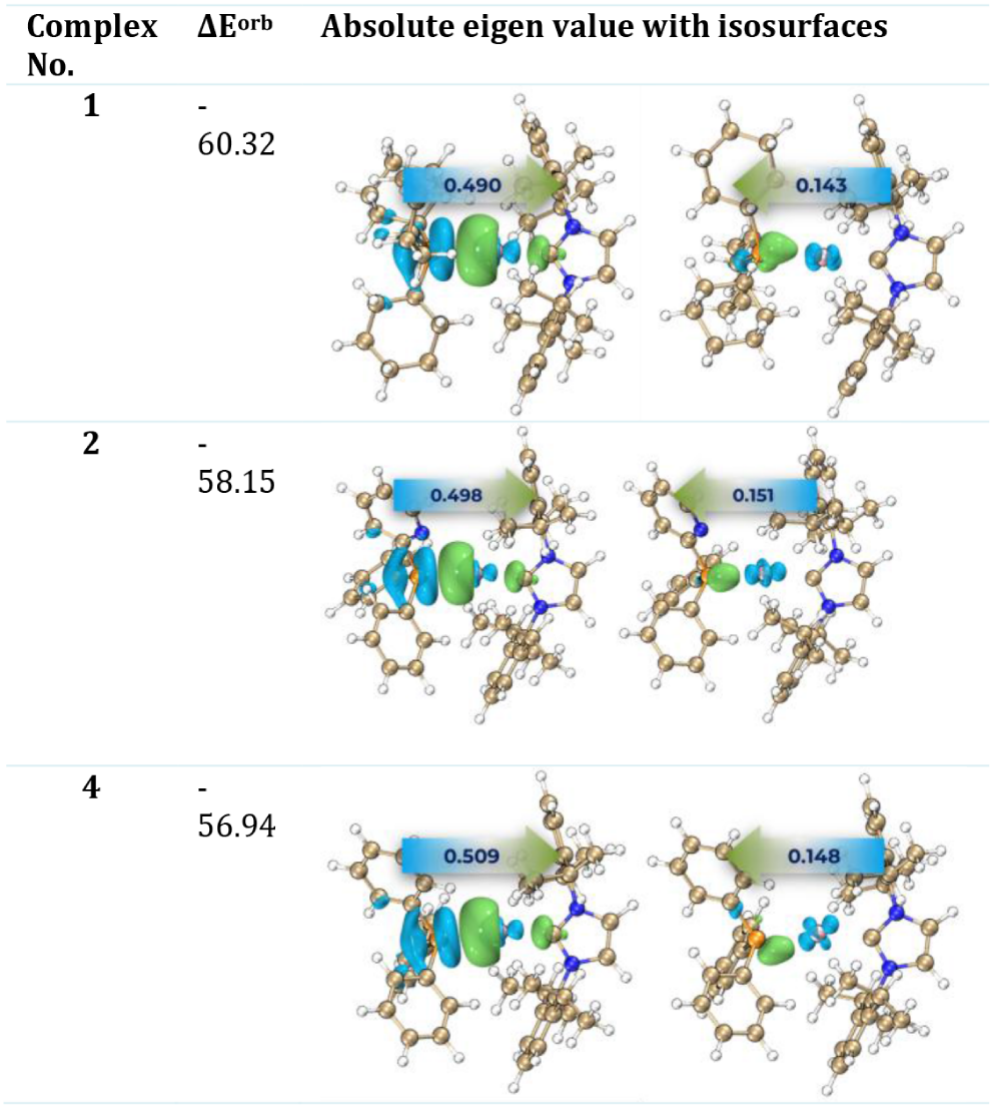
ETS-NOCV and Isosurfaces of Studied Complexes

**Table 3:** Comparison of Ag and Its Au Equivalents

Ag complexes	Δ*E* ^orb^	Au complexes	Δ*E* ^orb^
L–Ag–PCy_3_	–60.32	L–Au–PCy_3_	–107.54
L–Ag–PPh_2_Py	–58.15	L–Au–PPh_2_Py	–103.05
L–Ag–PPh_3_	–56.94	L–Au–PPh_3_	–102.35

## Conclusion

To a limited extent, (NHC)­Ag–Cl can be used as a precursor to produce [(NHC)­Ag-PR_3_]^+^ complexes. The type of solvent and the sequence of addition of reactants play an important role in the outcome of the derivatization reactions of (NHC)­Ag–Cl type precursors. Unlike their gold­(I) counterparts, [(NHC)­Ag-PR_3_]^+^ complexes are more labile in solution, particularly with PPh_3_ and bidentate ligands. The derivatization reactions led to the formation of unexpected silver­(I) complexes with only phosphine ligands, indicating that [(NHC)­Ag-PR_3_]^+^, if not stabilized by the phosphines, could easily decompose in solution, resulting in silver­(I)-phosphine complexes. A similar trend to (NHC)­gold­(I)-PR_3_ is observed in the HOMO–LUMO gap of silver couterparts. Different types of intramolecular noncovalent interactions in silver complexes are observed, with a particular focus on the interactions between the alkyl substituents of the phosphine ligand in **1** and the metal center. The calculated values obtained through ETS-NOCV analysis reveal an interesting trend that is comparable to the observed experimental results regarding the stability of these complexes in solution. In all complexes, the primary factor contributing to the orbital interactions arises from sigma-type interactions between the PR_3_ ligand and the silver metal center. Additionally, in all complexes, the effect of π-interaction in providing stability is negligible, in contrast to gold­(I) counterparts. This can primarily be attributed to the orbital mismatch between the filled d orbitals of the metal center and the σ* orbital from the PR_3_ ligand.

## Experimental Section

### General Considerations

Reagents and solvents were used as received from commercial suppliers. NMR spectra in solution were recorded on a Bruker DPX 400 MHz spectrometer in acetone-*d*_6_ or CDCl_3_ with SiMe_4_ (for ^1^H and ^13^C) and H_3_PO_4_ (for ^31^P) as external references. All complexation reactions were carried out under nitrogen gas. C, H, and N analyses were performed with a vario EL CHNS elemental analyzer.

### Synthesis of **1**, Method a

Tricyclohexylphosphine (32 mg, 0.11 mmol) was dissolved in methanol. Subsequently, ammonium hexafluorophosphate (55 mg, 0.11 mmol) was added to the reaction mixture, followed by the addition of IPr–Ag-Cl (60 mg, 0.11 mmol). The reaction proceeded for 10 min. Throughout this period, no white precipitate formed, and the reaction solution remained completely clear. Upon completion of the reaction, the solvent was fully evaporated, and the residue was dissolved in dichloromethane. The solution was then centrifuged and filtered through 20-μm filter paper to completely separate the potassium chloride byproduct. The solvent was evaporated again, and the residue was redissolved in chloroform. Finally, the product was purified by crystallization in a mixture of chloroform and *n*-hexane at 0 °C. Yield: 80 mg, 76%. ^1^H NMR (CDCl_3_, 400 MHz): δ 7.57–7.53 (m,1H, ArCH), 7.52–7.46 (m, 2H, ArCH), 7.36–7.34 (d, *J* = 8.0 Hz, 2H, ArC*H*), 7.33–7.31 (d, *J* = 8.0 Hz, 2H, ArCH), 7.26 (s, 1H, ArCH), 7.16–7.10 (m, 1H, ArCH), 2.57 (sept, *J* = 8.0 Hz, 4H, CH_iPr_), 2.12–2.02 (m, 2H, C_cyclohexyl_–H), 2.01–1.84 (m, 8H, C_cyclohexyl_–H), 1.84–1.72 (m, 2H, C_cyclohexyl_–H), 1.72–1.64 (m, 4H, C_cyclohexyl_–H), 1.64–1.57 (m, 5H, C_cyclohexyl_–H), 1.54–1.39 (m, 7H, C_cyclohexyl_–H), 1.30 (d, *J* = 8.0 Hz, 12H, CH_3‑iPr_), 1.25 (d, *J* = 8.0 Hz, 6H, CH_3‑iPr_), 1.24 (d, *J* = 8,0 Hz, 6H, CH_3‑iPr_), 1.21–1.05 (m, 2H, C_cyclohexyl_–H), 1.05–0.98 (m, 1H, C_cyclohexyl_–H), and 0.98–0.83 (m, 1H, C_cyclohexyl_–H). ^13^C NMR (101 MHz, CDCl_3_) δ 184.3 (d, *J*_C‑_^109^_Ag_ = 272 Hz, *J*_C‑_^107^_Ag_ = 242 Hz), 145.8 (ArC), 145.6 (ArC), 134.5 (ArC), 131 (ArC), 130.7 (ArC), 124.3 (ArC), 124.2 (ArC), 123.7 (ArC), 31.40 (C_aliphatic_), 31.1 (C_aliphatic_), 28.7 (C_aliphatic_), 26.8 (C_aliphatic_), 26.7 (C_aliphatic_), 26.2 (C_aliphatic_), 26.1 (C_aliphatic_), 26.0 (C_aliphatic_), 25.5 (C_aliphatic_), 24.9 (C_aliphatic_), 24.8 (C_aliphatic_), 24.5 (C_aliphatic_), 24.0 (C_aliphatic_). ^31^P NMR (162 MHz, CDCl_3_) δ 42.15 (d, *J*_P‑_^109^_Ag_ = 204 Hz, *J*_C‑_^107^_Ag_ = 180 Hz), −144.3. Anal. Calcd. for C_45_H_69_AgN_2_P_2_F_6_; C, 58.63; H, 7.54; N, 3.04. Found: C, 58.20; H, 7.01; N, 3.0.

### Synthesis of **2**

Similar procedure described for **2** was employed. Quantities of reactants: IPr–Ag–Cl (60 mg, 0.11 mmol), ammonium hexafluorophosphate (55 mg, 0.11 mmol), PPh_2_Py (30 mg, 0.11 mmol). Yield: 84 mg, 80%. ^1^H NMR (CDCl_3_, 400 MHz): δ 7.81–7.73 (m, 1H, ArCH), 7.64–7.56 (m, 2H, ArCH), 7.55–7.48 (m, 3H, ArCH), 7.46–7.41 (m, 3H, ArCH*)*, 7.40–7.33 (m, 8H, ArCH), 7.17–6.97 (m, 5H, ArCH), 2.59 (sept, 4H, CH_iPr_), 1.29 (d, 12H, J = 8.0 Hz, CH_3‑iPr_), 1.20 (d, 12H, *J* = 8.0 Hz, CH_3‑iPr_). ^13^C­{^1^H} NMR (CDCl_3_, 101 MHz) δ 133.5 (ArC), 132.5 (ArC), 129.5 (ArC), 128.0 (ArC), 125.4 (ArC), 30.1 (C_aliphatic_), 29.5.5 (C_aliphatic_), 26.6 (C_aliphatic_), 22.2 (C_aliphatic_). Carbene carbon atom (NCN) was not observed. ^31^P NMR (162 MHz, CDCl_3_) δ 17.1–144.4. P–Ag­(I) coupling was not observed. Anal. Calcd. for C_44_H_50_AgN_3_P_2_F_6_; C, 58.41; H, 5.57; N, 4.64. Found: C, 58.20; H, 5.25; N, 4.3.

### Synthesis of **4**

Triphenylphosphine (30 mg, 0.11 mmol) was dissolved in acetone, after which potassium hexafluorophosphate (62 mg, 0.11 mmol) was added to the reaction mixture, followed by the addition of IPr–Ag–Cl (60 mg, 0.11 mmol). A white precipitate formed in the reaction vessel after 5 min. The reaction continued for an additional 10 min. Subsequently, the solvent was evaporated, and the residue at the bottom of the reaction flask was dissolved in chloroform. After centrifugation, the solution was filtered through 20-μm filter paper. Finally, the product was purified by crystallization in a chloroform and ether mixture at 0 °C. Yield: 75 mg, 72%. ^1^H NMR (CDCl_3_, 400 MHz): δ 7.64–7.56 (m, 3H, ArCH), 7.55–7.48 (m, 3H, ArCH), 7.46–7.41 (m, 3H, ArCH), 7.33–7.17 (m, 9H, ArCH), 7.17–6.97 (m, 5H, ArCH), 2.59 (sept., *J* = 8.0 Hz, 4H, CH_iPr_), 1.29 (d, *J* = 8.0 Hz, 12H, CH_iPr_), 1.21 (d, *J* = 8.0 Hz, 12H, CH_3‑iPr_). ^31^P NMR (162 MHz, CDCl_3_) δ −18.8 (t, *J*_Ag–P_ = 972 Hz), −144.4. Anal. Calcd. for C_45_H_51_AgN_2_P_2_F_6_; C, 59.81; H, 5.69; N, 3.10. Found: C, 59.01; H, 5.15; N, 3.01.

### X-ray Crystal Structure Determination and Refinement

The X-ray diffraction data of **1** and **5** were collected at 95 K with Rigaku OD Supernova diffractometer using Atlas S2 CCD detector and mirror-collimated radiation from a sealed microfocus X-ray tube (λ = 1.54180 Å for **1** and **5**). The X-ray diffraction data of **2** and **7** were collected at 120 K with Rigaku OD Gemini diffractometer using Atlas S2 CCD detector and a classical sealed X-ray tube with graphite monochromator, λ = 0.71073 Å. Integration of the CCD images, absorption correction and scaling were done by the program CrysAlisPro 1.171.41.123a (Rigaku Oxford Diffraction, 2022). Crystal structures were solved by charge flipping with the program SUPERFLIP,^71^ and structure **1** was refined using Crystals^72^ while structures **2**, **5** and **7** were refined with the Jana2020.^73^ The hydrogen atoms were discernible in residual electron density maps and could be refined to reasonable geometry, but according to common practice, they were kept at ideal positions with *U*_iso_ kept at 1.2 *U*_eq_(C). The molecular structure plots were prepared with Mecrucry 3.0.^74^ Crystallographic data, details of the data collection, structure solution and refinements are listed in Table S1.

## Supplementary Material



## Notes

The geometries are provided separately in a file labeled GEOMs.xyz. Availability of crystallographic data: 10.57680/asep.0600481. The Supporting Information is available free of charge at https://pubs.acs.org/doi/10.1021/acsomega.5c07921.

The spectroscopic data for compounds **1**–**4**, along with Table S1, are included in the Supporting Information section as the manuscript SI (PDF)

## Abbreviations

IPr: *N*,*N*-bis­(2,6-diisopropylphenyl)­imidazol-2-ylidene
dppm: Bis­(diphenylphosphino)­methane
dppe: 1,2-bis­(diphenylphosphino)­ethane
dppf: 1,1’-bis­(diphenylphosphino)­ferrocene
PCy_3_: Tricyclohexylphosphine
PPh_3_: Triphenylphosphine
PPh_2_Py: Diphenyl-2-pyridylphosphine
S­(S)­P­(OR)_2_: dialkyldithiophosphate
