# Correction to “N-Heterocyclic Carbene-Ag(I)-Phosphine Complexes: Comprehensive Synthesis, Characterization, and Bonding Analysis via Density Functional Theory”

**DOI:** 10.1021/acsomega.6c04324

**Published:** 2026-07-17

**Authors:** Abdollah Neshat, Mohammad Reza Yousefshahi, Mahdi Cheraghi, Vaclav Eigner, Michal Dusek

Changes:

The geometries are provided separately in a file labeled GEOMs.xyz. Availability of crystallographic data: 10.57680/asep.0600481

To:

The geometries are provided separately in a file labeled GEOMs.xyz. Availability of crystallographic data: 10.57680/asep.0648479

