# Supplementary material for: N‑Heterocyclic Carbene-Ag(I)-Phosphine Complexes: Comprehensive Synthesis, Characterization, and Bonding Analysis via Density Functional Theory
Source: ACS Omega. 2026 Feb 20;11(9):14316–26. doi: 10.1021/acsomega.5c07921 (PMC12980218; doi:10.1021/acsomega.5c07921)
Supplement: Supplementary file 1 [file ao5c07921_si_001.pdf]

Supporting Information

N-Heterocyclic Carbene-Ag(I)-Phosphine Complexes: Comprehensive Synthesis, Characterization, and Bonding Analysis via Density Functional Theory

Abdollah Neshat,*† Mohammad Reza Yousefshahi,† Mahdi Cheraghi,† Vaclav Eigner,*‡ Michal Dusek,‡

† Department of Chemistry, Institute for Advanced Studies in Basic Sciences (IASBS), 444 Prof. Sobouti Blvd., Gava Zang, Zanjan 45137-66731, Iran, a.neshat@iasbs.ac.ir

‡ Institute of Physics of the Czech Academy of Sciences, Na Slovance 2, 18221 Prague 8, The Czech Republic

Table of Contents

Figure S1. ^{1}H NMR spectrum of 1..……………..…………………………………………....S3

Figure S2. ^{13}C NMR spectrum of 1…………………………………………………...S4

Figure S3. ^{31}P NMR spectrum of 1. ……………………………………………………..S5

Figure S4. ^{1}H NMR spectrum of 2, ……………………..……………………………………S6

Figure S5. ^{13}C NMR spectrum of 2………………………………………………………...S7

Figure S6. ^{31}P NMR spectrum of 2,.……………………...……………………………...S8

Figure S7. ^{1}H NMR spectrum of 3,.………………………………………….......................S9

Figure S8. ^{1}H NMR spectrum of reaction of 4. ……………………………………………..S10

Figure S9. ^{31}P NMR spectrum of 4…………………………………………………………..S11

Figure S10. ^{1}H NMR spectrum of reaction 6………...…………………………………..….S12

Figure S11. ^{1}H NMR spectrum of reaction of 7……………………………………………….S13

Table S1. Crystal and structure refinement data of 1, 2, 5, and 7………………………………S14

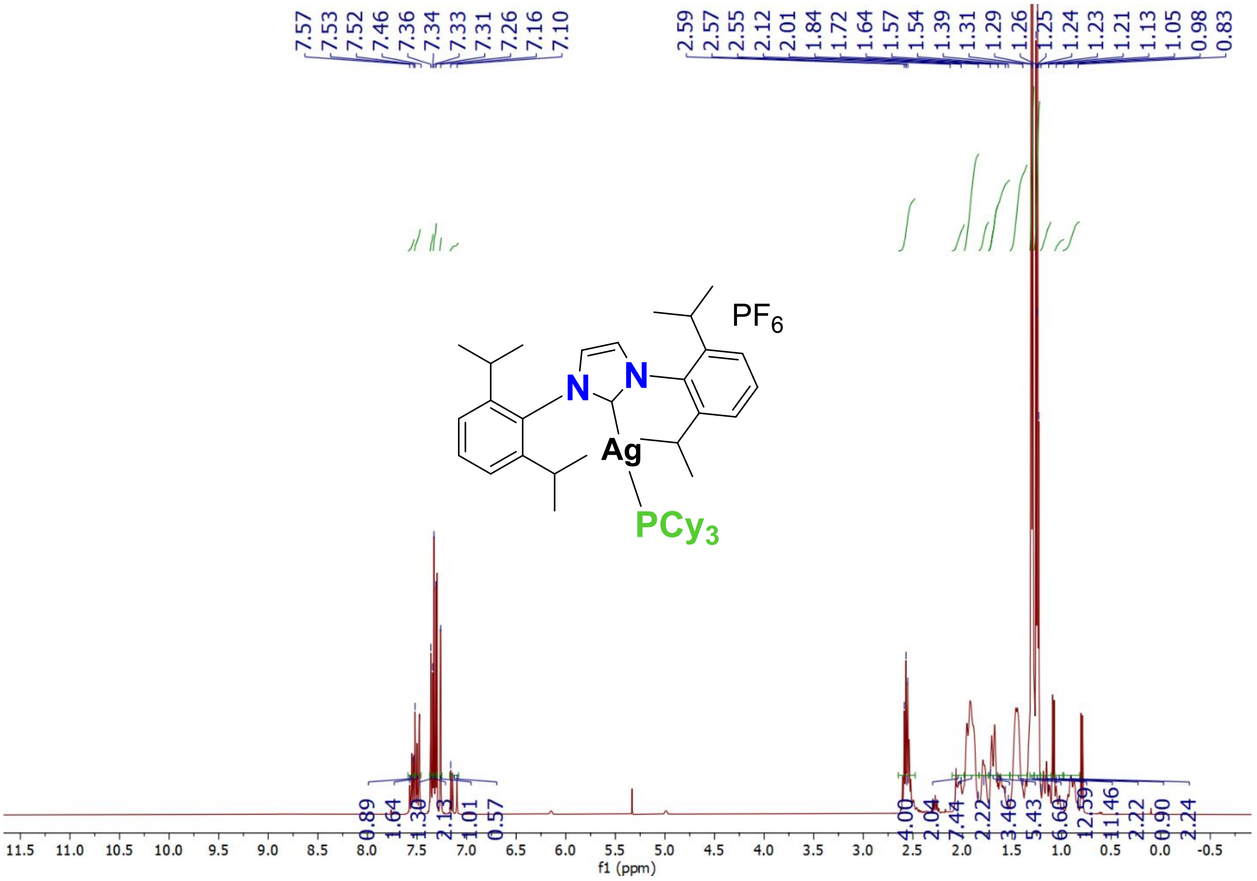

Figure S1. ^{1}H NMR spectrum of **1** in $CDCl_3$. A signal at ~5.3 ppm belongs to traces of dichloromethane.

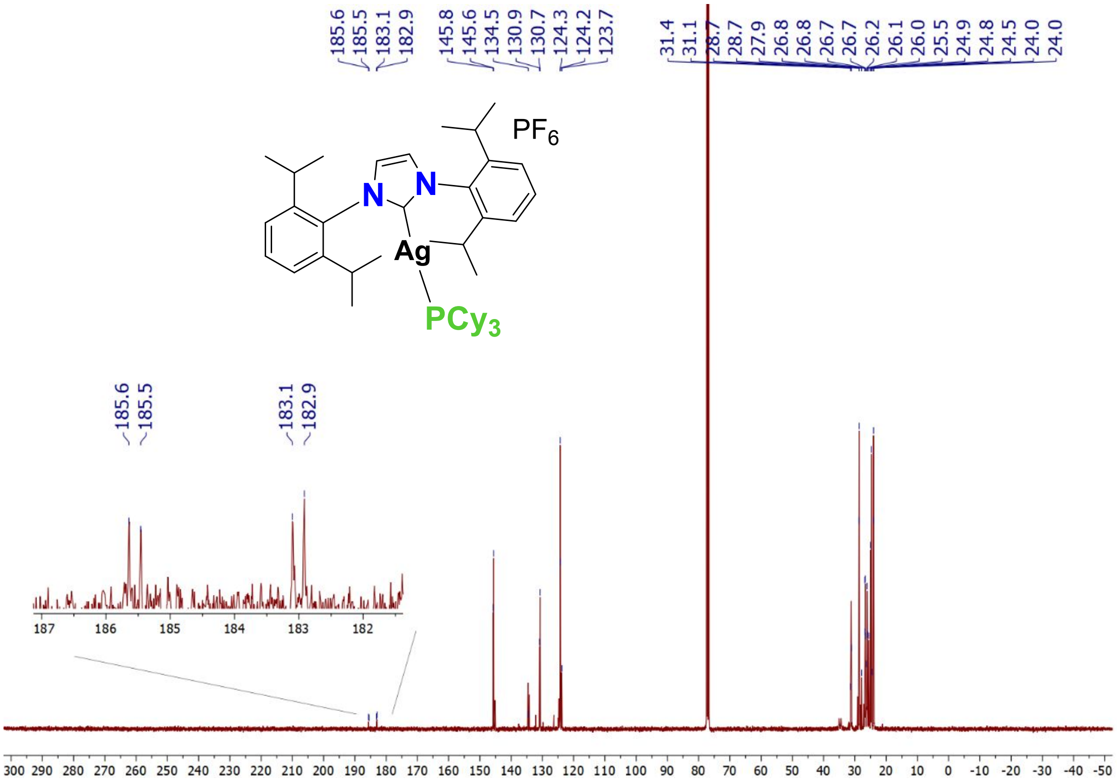

Figure S2. ^{13}C NMR spectrum of **1**. Signals at ~78 ppm belong to chloroform.

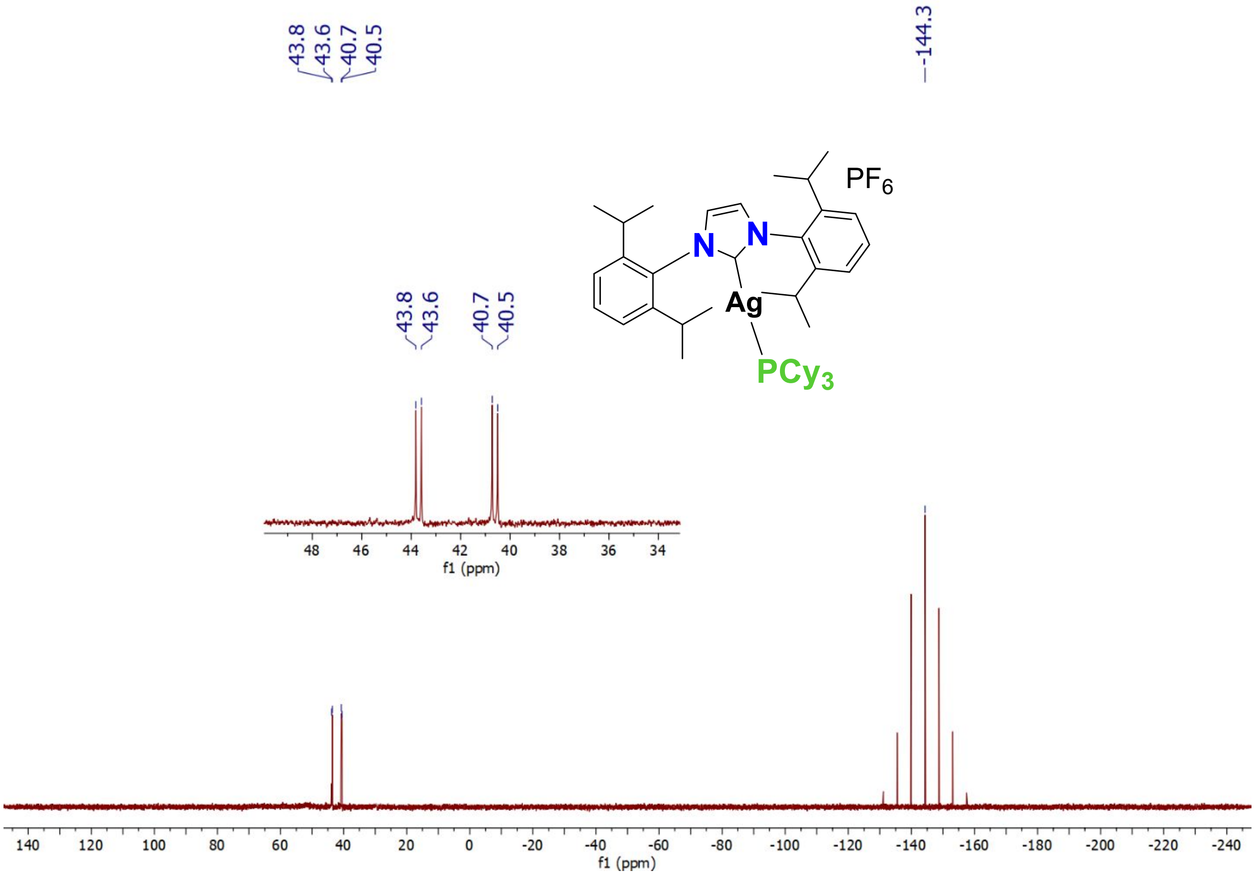

Figure S3. ^{31}P NMR spectrum of **1**.

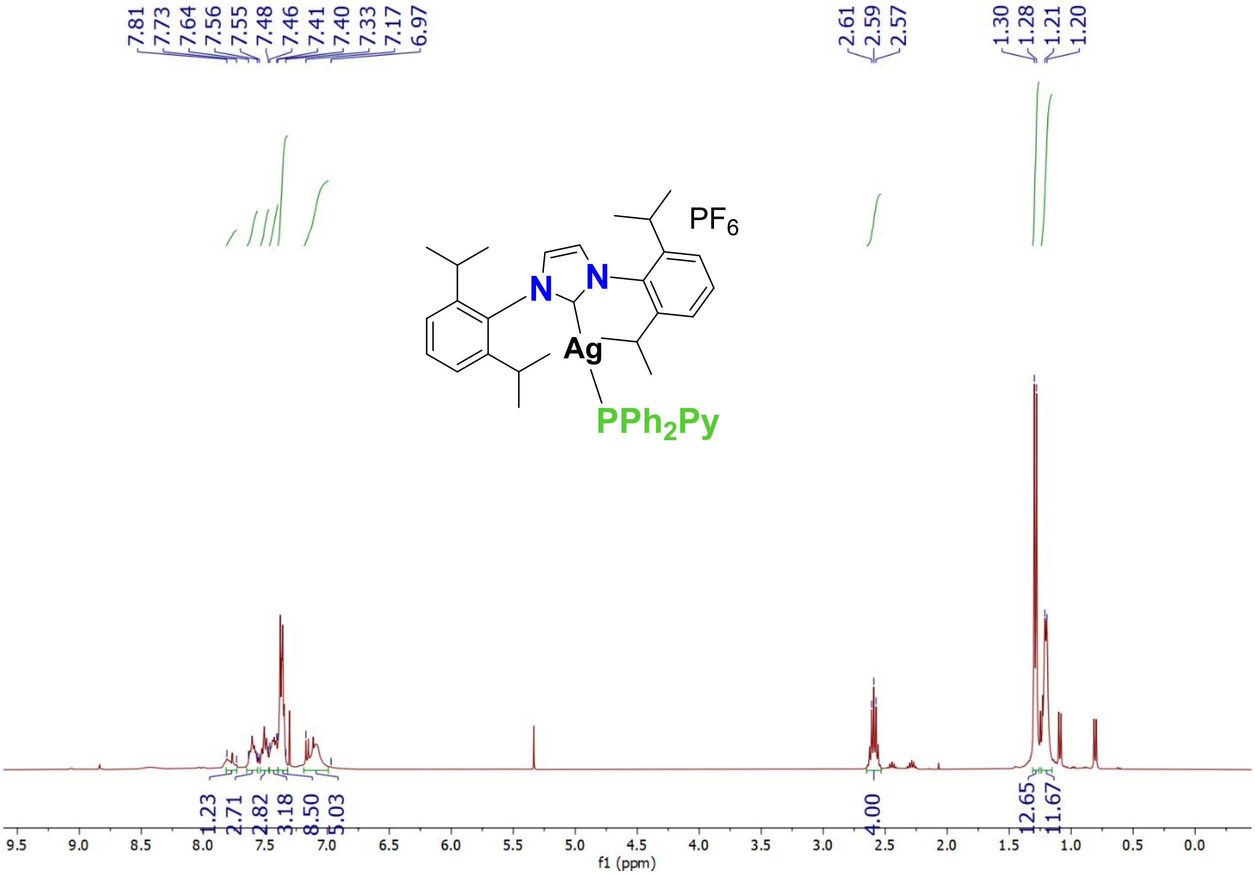

Figure S4. ^{1}H NMR spectrum of **2**. Peaks below 1 ppm denote $[IPrAg]^{+}$ species.[1] A peak at ~5.3 ppm belongs to traces of dichloromethane.

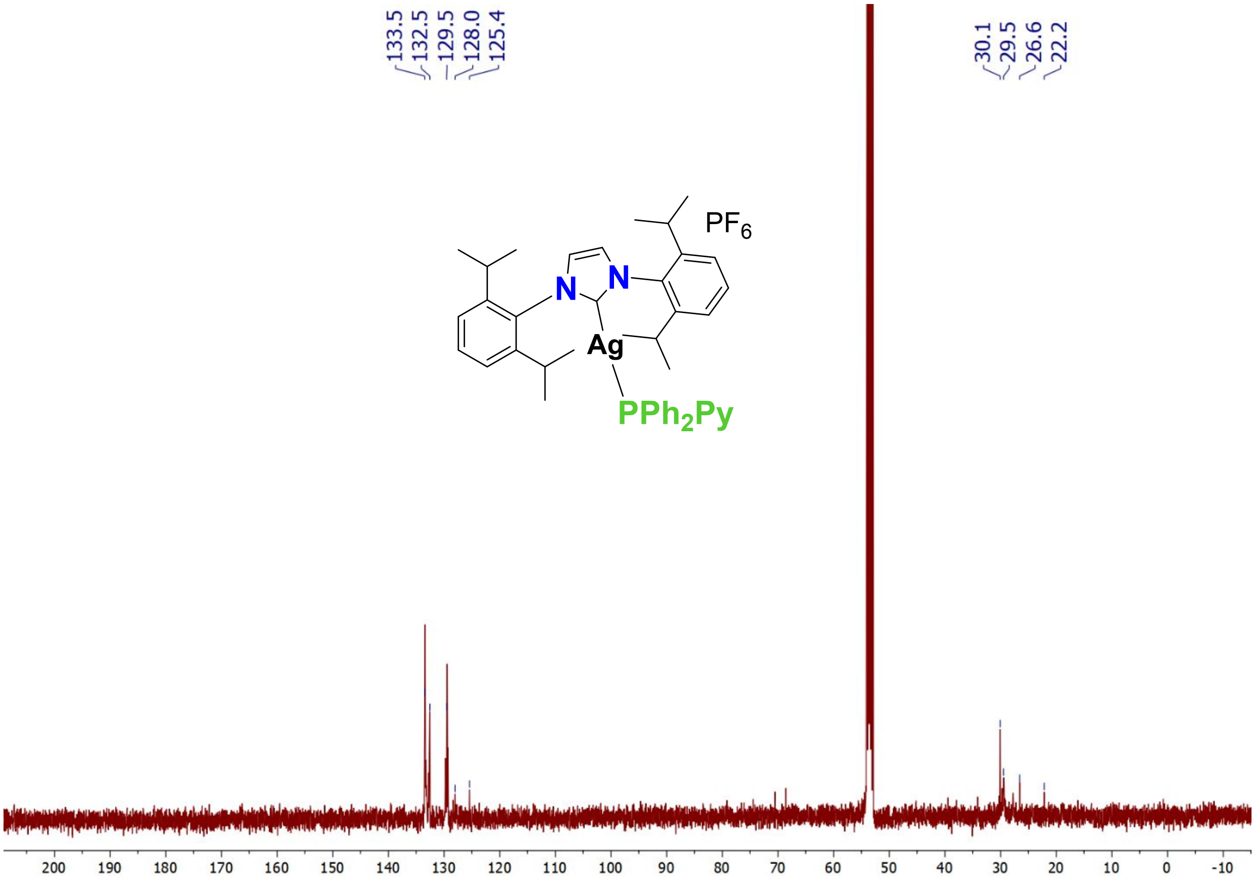

Figure S5. ^{13}C NMR spectrum of **2**. Multiplet signal at ~53 ppm belong to CD_2Cl_2.

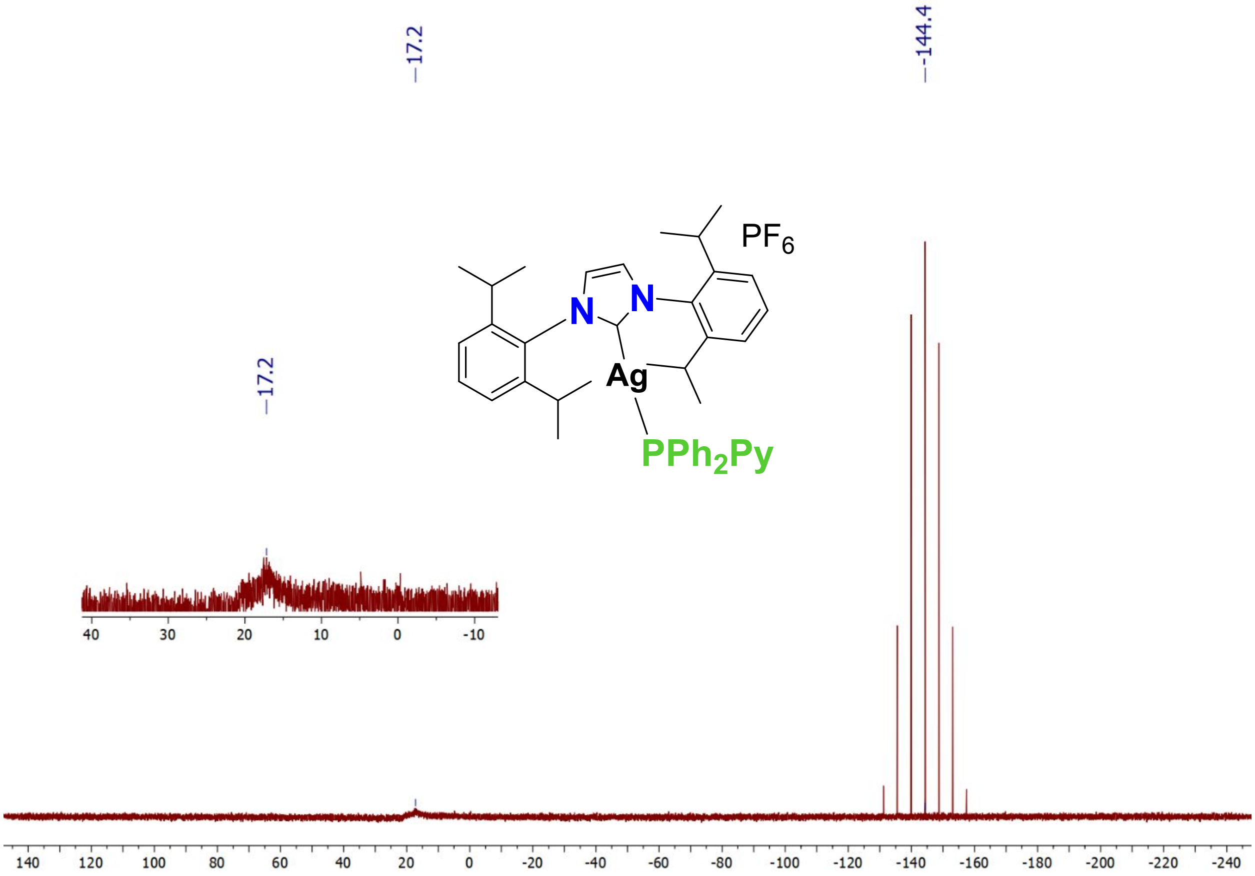

Figure S6. ^{31}P NMR spectrum of **2**.

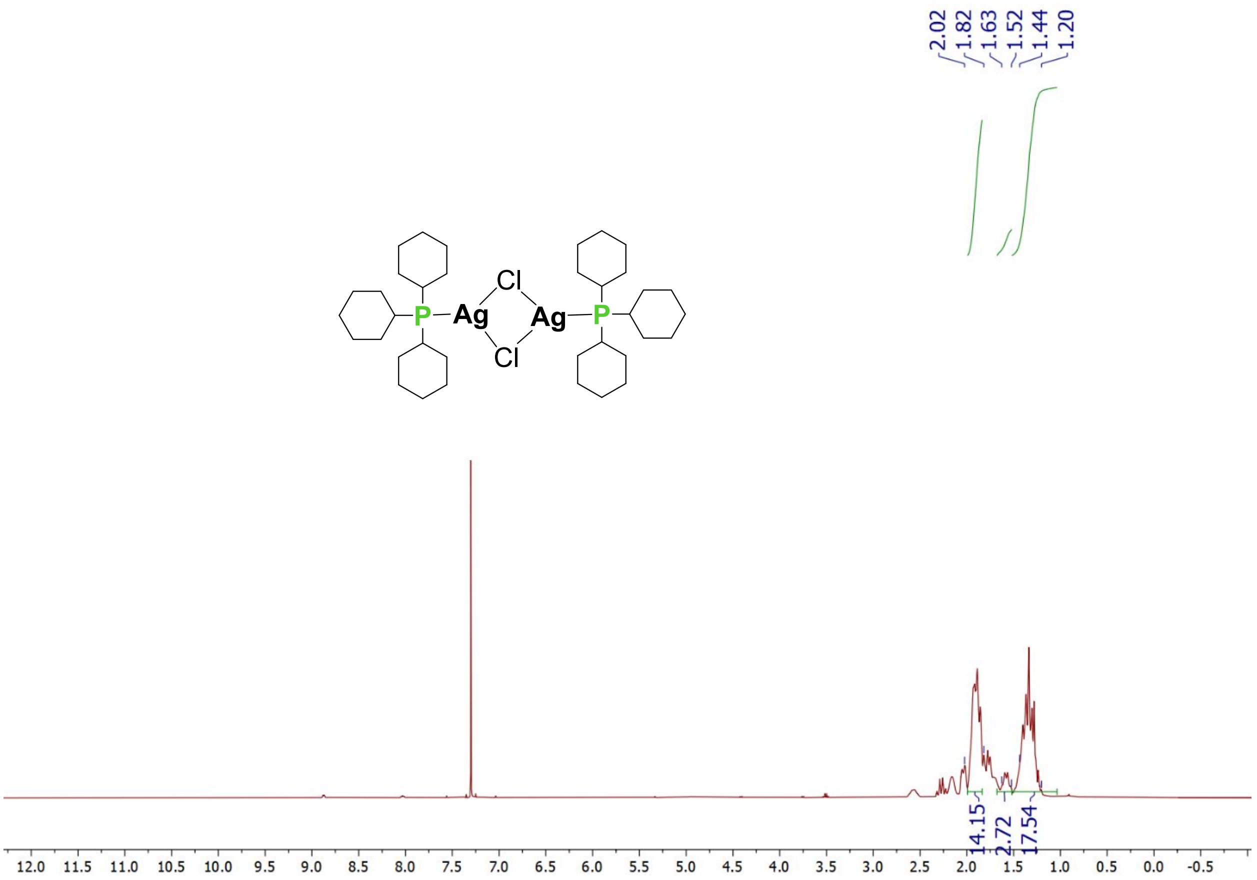

Figure S7. ^{1}H NMR spectrum of reaction of **3**.

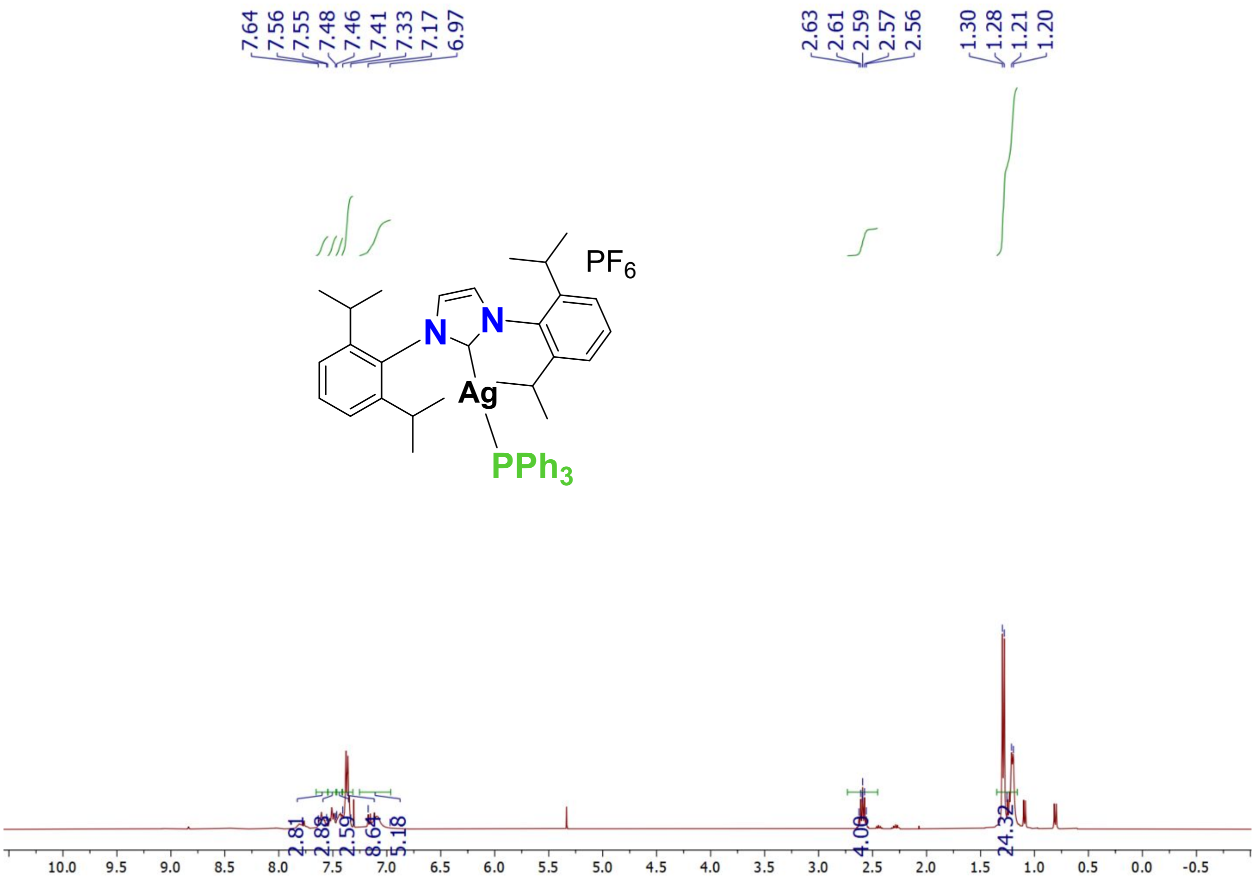

Figure S8. ^{1}H NMR spectrum of reaction of **4**. Peaks below 1.1 and 0.85 ppm denote $[IPrAg]^+$ species.[1] A signal at ~5.3 ppm belongs to traces of dichloromethane.

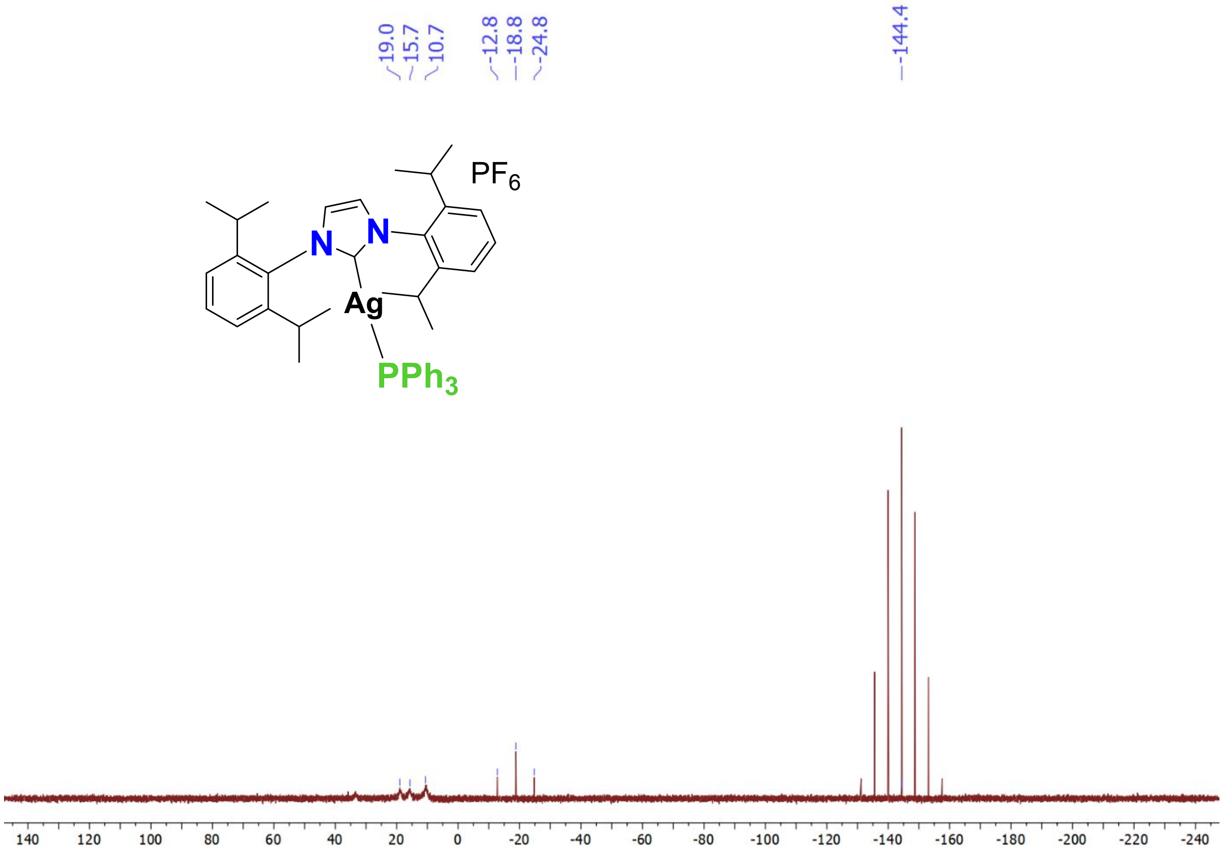

Figure S9. ^{31}P NMR spectrum of **4**.

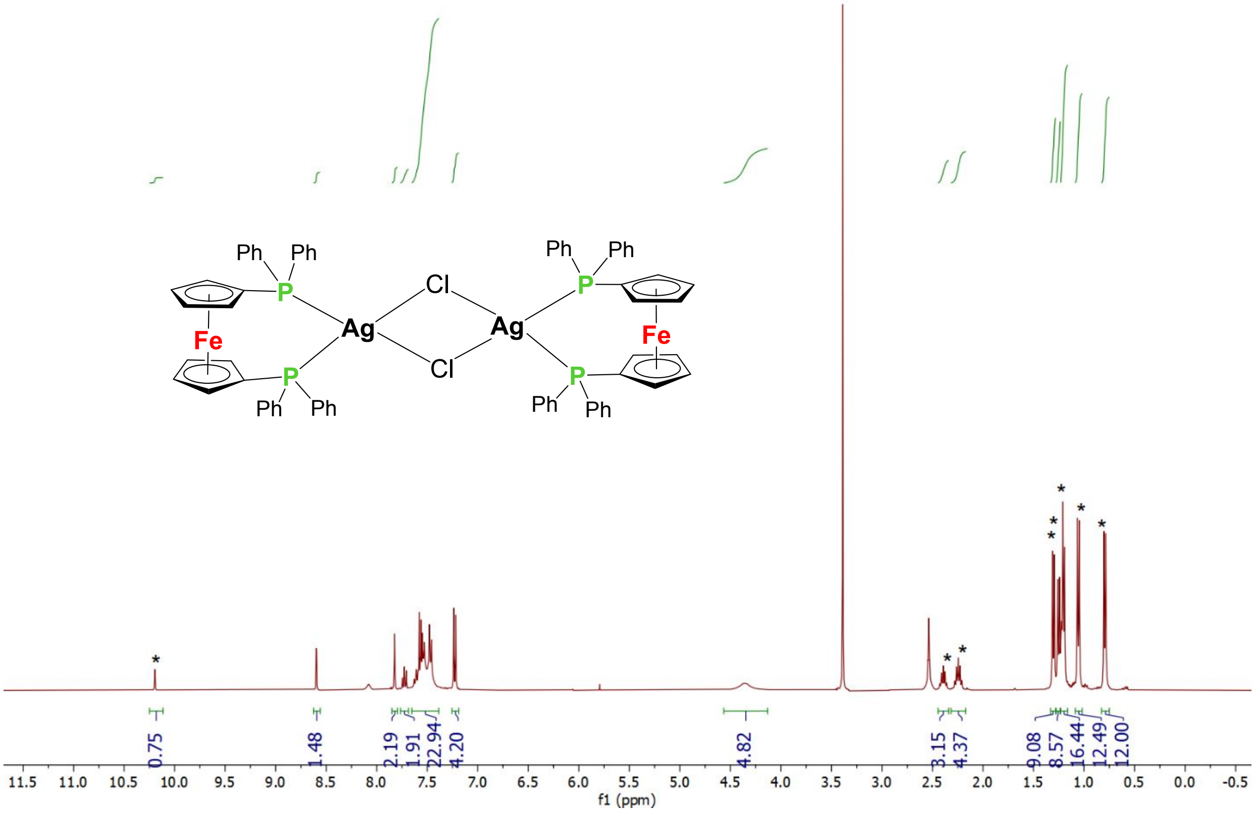

Figure S10. ^{1}H NMR spectrum of reaction mixture of **6**. Stars denote formation of IPr.PF_6 and [IPr_2.Ag]PF_6.

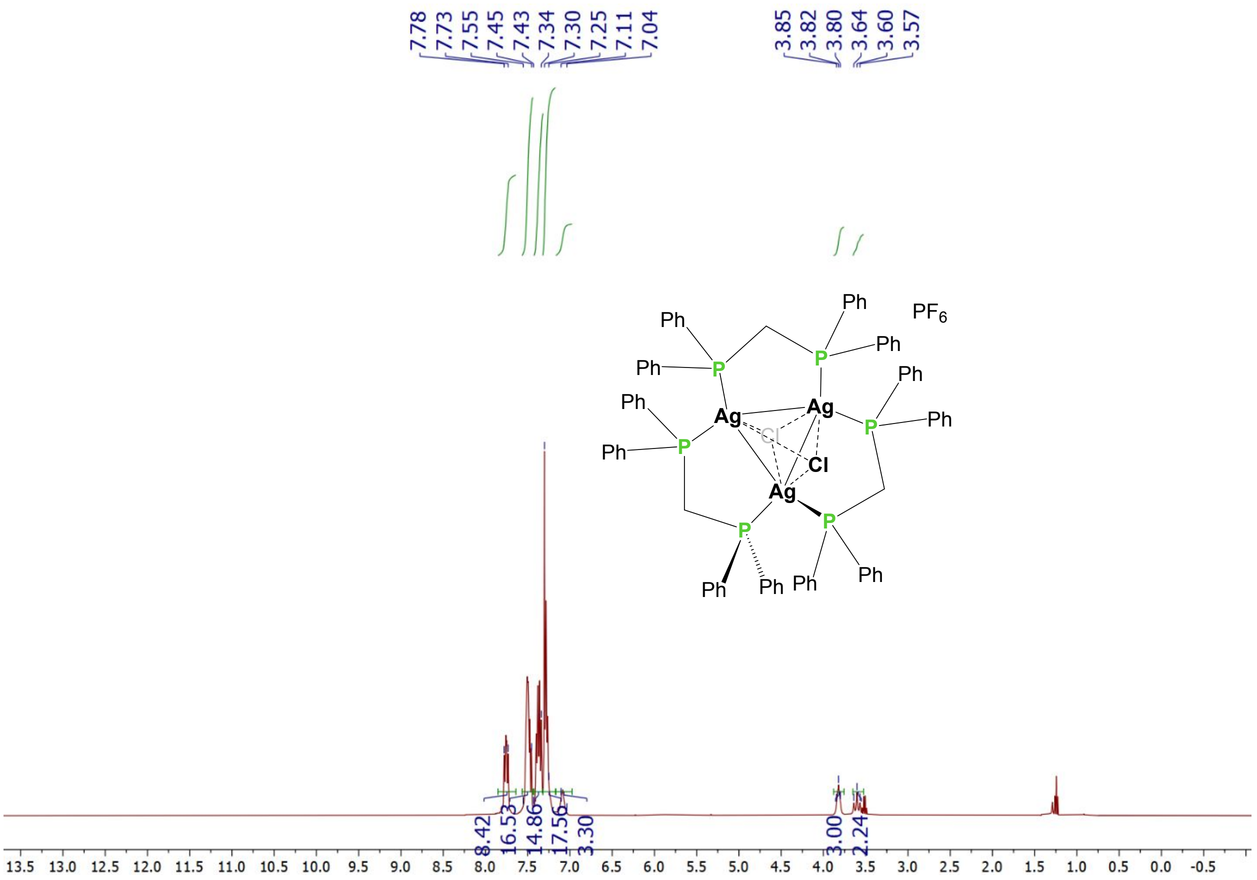

Figure S11. ^{1}H NMR spectrum of reaction of **7**.

Table S1. Crystal and structure refinement data of **1**, **2**, **5**, and **7**.

Identification code	**1**	**2**	**5**	**7**
Empirical formula	$C_{92}H_{140}AgCl_6F_{12}KN_4P_4$	$C_{44}H_{50}AgF_6N_3P_2$	$C_{73}H_{61}Ag_1Cl_3F_6P_5$	$C_{75}H_{66}Ag_3Cl_2F_6P_7$
Formula weight	2013.70	904.7	1421.4	1692.7
T, K	95	120	95	120
λ, Å	1.54184	0.71073	1.54184	0.71073
Crystal system	orthorhombic	tetragonal	monoclinic	triclinic
Space group	$Pca2_1$	$P4_3$	$P2_1/c$	P-1
a, Å	35.9255(4)	11.9253(2)	11.9965(3)	15.4218(5)
b, Å	17.0795(2)	11.9253(2)	22.9339(6)	15.6552(6)
c, Å	16.1792(2)	30.0628(7)	23.2761(5)	16.4766(6)
α, °	90	90	90	101.855(3)
β, °	90	90	90.813(2)	107.204(3)
γ, °	90	90	90	98.158(3)
V, Å^3	9927.4(2)	4275.28(14)	6403.2(3)	3630.5(2)
Z	4	4	4	2
ρ_{calc}, g cm^{-3}	1.347	1.4056	1.4744	1.5484
μ, mm^{-1}	4.657	0.608	5.361	1.087
F(000)	4223.993	1864	2904	1700
Crystal size, mm	0.060 × 0.088 × 0.135	0.398 × 0.191 × 0.08	0.065 × 0.058 × 0.039	0.28 × 0.073 × 0.031
θ_{min}, θ_{max}, °	3.54, 73.24	2.42, 29.38	3.68, 73.5	2.58, 29.48
Index ranges	0<=h<=44, 0<=k<=21, −20<=l<=16	−16<=h<=15, −16<=k<=16, −40<=l<=39	−14<=h<=14, −19<=k<=28, −28<=l<=28	−19<=h<=21, −21<=k<=21, −21<=l<=22
Reflections collected	16874	78740	25384	33247
Independent reflections, R_{int}	16870, 0.00	11066, 0.0687	25244, 0.0	16905, 0.0374
θ_{full}, ° (completeness 98%)	69.132	29.28	73.5	27.14
Data/restraints/parameters	16870/38/1093	11066/0/506	25244/11/825	16905/0/838
Goodness-of-fit on F^2	0.9838	1.2923	2.0402	1.0206
R_1 [I>3σ(I)]*	0.0738	0.0345	0.0692	0.0371
wR_2 [I>3σ(I)]*	0.2040	0.0643	0.1552	0.0689
R_1 (all data)	0.0821	0.0457	0.0899	0.0698
wR_2 (all data)	0.2180	0.0680	0.1590	0.0829
$\Delta\rho_{min}$, $\Delta\rho_{max}$, e Å^{-3}	-1.80, 2.02	-0.29, 0.22	-1.14, 2.65	-0.26, 0.40

Reference

(1) Gibard, C.; Fauché, K.; Guillot, R.; Jouffret, L.; Traïkia, M.; Gautier, A.; Cisnetti, F. Access to Silver-NHC Complexes from Soluble Silver Species in Aqueous or Ethanolic Ammonia. J. Organomet. Chem. 2017, 840, 70–74. https://doi.org/https://doi.org/10.1016/j.jorganchem.2017.04.009.
